# Effectiveness of Telerehabilitation Exercise Programme on Disability and Pain in Patients With Chronic Non‐Specific Neck Pain: Randomised Controlled Trial Assessor‐Blinded

**DOI:** 10.1002/msc.70119

**Published:** 2025-05-15

**Authors:** Juliene Corrêa Barbosa, Bruna Vale da Luz, Breno Felipe Portal da Silva, Amelia Pasqual Marques, Bruno Tirotti Saragiotto, Josielli Comachio, Mauricio Oliveira Magalhaes

**Affiliations:** ^1^ Master's Program in Human Movement Sciences Federal University of Pará Belém‐Pará Brazil; ^2^ Faculty of Physiotherapy and Occupational Therapy Federal University of Pará Belém‐Pará Brazil; ^3^ Department of Physiotherapy Rehabilitation Sciences Program Speech‐Language Pathology and Audiology and Occupational Therapy Faculty of Medicine University of São Paulo São Paulo Brazil; ^4^ Master's Programs in Physical Therapy Universidade Cidade de São Paulo São Paulo Brazil; ^5^ Sydney Musculoskeletal Health School of Health Sciences Charles Perkins Centre Faculty of Medicine and Health University of Sydney Sydney Australia

**Keywords:** chronic pain, exercise therapy, neck pain, telehealth, telerehabilitation

## Abstract

**Introduction:**

Chronic neck pain is an important public health problem. Telerehabilitation has emerged as an important tool for individuals with musculoskeletal conditions.

**Objectives:**

The study aims to identify the effectiveness of a telerehabilitation exercise programme compared with a digital self‐care booklet on non‐specific neck pain.

**Methods:**

A randomised controlled trial assessor‐blinded, 3 months follow‐up. 70 patients were randomised into two groups of 35. The telerehabilitation group received 6 weeks of individualised training through vídeo calls and an online booklet. The control group received an online booklet. The primary outcome was functional disability. Secondary outcomes included pain intensity, global perceived effect, self‐efficacy, quality of life, and kinesiophobia. All outcomes were assessed at baseline, 6 weeks, and a 3‐month follow‐up.

**Results:**

There was a significant difference between groups for functional disability (Mean 10.3, CI 95% 4.8–15.7), pain intensity (Mean 2.8, CI 95% 1.4–4.1), global perceived effect (Mean −2.3, CI 95% −3.7 to −0.9), and self‐efficacy (Mean −24.7, CI 95% −41.0 to −8.4) at the 6‐week. At the 3‐month follow‐up, statistically significant differences were observed for perceived overall effect (Mean −2.0, CI 95% −3.4 to −0.6) and self‐efficacy (Mean −26.3, CI 95% −42.8 to −9.8).

**Conclusions:**

Telerehabilitation is effective in improving disability and pain intensity compared with self‐care booklets only in individuals with non‐specific chronic neck pain.

**Trial registration:**

This trial is registered at https://ensaiosclinicos.gov.br/rg/RBR‐10h7khvk under the registration number RBR10h7khvk at 09/16/2022.

## 1 Introduction

Chronic neck pain is characterised as a disabling condition that causes difficulty in performing professional, social, and sports activities (de et al. 2018) and is an important public health problem with high levels of disability worldwide (Fandim et al. 2020). Neck pain is classified as specific, neuropathic and non‐specific, the latter being when it cannot be attributed to a specific cause (Fandim et al. 2020). It is estimated that chronic neck pain affects more than 65 million people annually worldwide, with annual incidence rates ranging from 10.4% to 21.3% (Hoy et al. 2010). Additionally, according to the Global Burden of Disease 2019, chronic neck pain is ranked 11th out of the 369 conditions in terms of years lived with disability (YLDs) (James et al. 2018).

Neck pain has a favourable initial clinical course. The natural course of the disease is associated with a better clinical prognosis with cases resolving spontaneously within a few weeks and months; however, recurrence rates continue to be high and there is a significant burden generated on patients over time (Cohen 2015; Fandim et al. 2021). Conservative treatment is recommended as an effective approach for chronic neck pain according to recent treatment guidelines (Blanpied et al. 2017; Corp et al. 2021; Fillipo et al. 2022). This includes exercises, such as strengthening exercises, motor control, and proprioceptive training, stretching, mobilisations, and manipulations (Blanpied et al. 2017; Corp et al. 2021; Fillipo et al. 2022).

To bridge the gap in access to these recommended conservative treatments, telerehabilitation has emerged as an important tool, especially during the COVID‐19 pandemic, promoting significant improvements in pain intensity and functional disability in individuals with musculoskeletal conditions (Alsobayel et al. 2022; Tuckson et al. 2017). The use of telecommunication tools such as mobile applications, video conferences, virtual reality, and sensors has seen a surge, thereby facilitating improved access to healthcare services (Russell 2007). This growth, noticed over the past year, has played a critical role in decreasing costs and time for patients and healthcare professionals (Russell 2007).

The convenience and flexibility of telerehabilitation have contributed to increased healthcare accessibility and greater adherence to exercise programs using video conferences, video games and telephone counselling/coaching (F. Costa et al. 2022; Wang et al. 2019; Yeo et al. 2021). This progress has demonstrated notable effectiveness in addressing various health conditions, including musculoskeletal pain, osteoarthritis, and low back pain (Seron et al. 2021). However, few studies have investigated the effectiveness of telerehabilitation in individuals with chronic nonspecific neck pain (Gialanella et al. 2017; Kosterink et al. 2010; Özel and Kaya Ciddi 2022). Moreover, these studies present relevant methodological limitations, such as the absence of evaluator blinding, intention‐to‐treat analysis, and non‐concealed participant allocation, which takes low certainty of evidence in these studies. Thus, the effectiveness of telerehabilitation in patients with nonspecific chronic neck pain remains unclear. Robust methodological studies of high quality are imperative to elucidate the effectiveness of telerehabilitation in this population.

Therefore, this study aimed to identify the effectiveness of a telerehabilitation exercise programme compared with a digital self‐care booklet immediately and in the short‐term follow‐up on non‐specific neck pain.

## 2 Methods

### 2.1 Design

This was a two‐arm randomised controlled trial with concealed allocation, blinded assessor, and intention‐to‐treat analysis and reported in accordance with Consolidated Standards of Reporting Trials (CONSORT) (Schulz et al. 2010) and Standard Protocol Items: Recommendations for Interventional Trials (SPIRIT) (Chan et al. 2013). Participants were recruited from a waiting list at a Rehabilitation Centre via social media platforms (Instagram, Facebook, and local university websites). All eligible individuals who agreed to participate in the study signed a consent form. The detailed protocol for this clinical trial was previously published (Barbosa et al. 2023).

Eligibility was initially determined by the blinded treatment assessor. Randomisation was conducted using Excel software in a 1:1 ratio by a researcher not involved in data collection, resulting in a randomly ordered list of allocation codes. To conceal the allocation list, the randomisation codes were placed in consecutively numbered sealed opaque envelopes (Herbert et al. 2005).

The data outcomes assessor, responsible for collecting outcome data, remained unaware of participant allocation. Due to the nature of the interventions, the therapist administering the interventions in the groups was not blinded to the randomisation. Participants were not informed about the specific treatment they received; however, given the nature of the interventions, this would not have been sufficient to blind the participants.

### 2.2 Participants

Participants were eligible if they had non‐specific chronic neck pain (duration > 12 weeks), were aged between 18 and 60 years, experienced a minimum pain intensity of 3 points on the 11‐point Pain Numerical Scale (L. O. P. Costa et al. 2008), and had access to the internet and a computer/or a smartphone (Bernal‐Utrera et al. 2019).

Individuals with severe musculoskeletal disorders, cardiovascular and metabolic disorders, history of neurological injuries, use of muscle relaxants, obesity (body mass index  >  30 kg/m^2^), and red flags (unexplained weight loss, fever, moderate to severe trauma), cognitive problems, visual or auditory impairments, or any health condition that impeded safe and suitable participation in online exercise sessions were also excluded (Almeida and Kraychete 2017).

## 3 Intervention

### 3.1 Telerehabilitation Group

Participants in the Telerehabilitation group consisted of a video conferencing exercise programme via the Google Meet platform, twice a week, for 45 min per session for 6 weeks delivered by a physiotherapist with over 2 years of professional experience.

The exercise protocol comprised three distinct phases: in the initial phase, emphasis was placed on enhancing mobility during the first and second weeks of treatment; the subsequent phase concentrated on specific exercises for neck and core stabilisation (weeks 3 and 4); finally, the third phase was dedicated to enhancing the endurance of both upper and lower limbs (weeks 5 and 6). Participants in these groups were instructed to incorporate weights into their exercises. A 1 kg weight was introduced in week 5 for sets of 12–15 repetitions, progressing to 2 kg in week 6. The sequence of the exercise programme protocol was carefully structured to optimise its effectiveness. The sequence of the exercise programme protocol was previously published (Barbosa et al. 2023).

Participants randomised to the experimental group also received educational material through a digital booklet containing self‐care information about chronic pain education, the benefits of exercise, and general advice on healthy lifestyle (e.g., sleep, and diet information).

### 3.2 Control Group

Participants in the control group received the same educational material (i.e., a digital booklet for self‐care). Additionally, participants received one text message at the beginning of the study through the WhatsApp application, SMS, or email to encourage the maintenance of healthy habits. Furthermore, telephone contact was made available during the study period for participants to address any questions related to the booklet.

### 3.3 Outcome Measures

All participants underwent a 6‐week treatment programme. Follow‐up measurements were conducted at 6 weeks and the 3‐month follow‐up.

#### 3.3.1 Primary Outcome

The primary outcome measure was disability assessed by the Brazilian version of the Neck Disability Index (Cook et al. 2006). This self‐administered questionnaire consists of 10 items that enquire about limitations in daily life activities due to neck pain. Scores on the questionnaire ranged from 0 to 50 points, with higher values indicating a greater level of disability (Cook et al. 2006).

#### 3.3.2 Secondary Outcomes

Secondary outcomes included pain intensity assessed by the Pain Numerical Rating scale (NRS) (i.e., 11 points (0–10), where 0 represents no pain and 10 the worst imaginable pain) (L. O. P. Costa et al. 2008); Global Perceived Effect (i.e., 11 items ranging from −5 “extremely worse” to 0 “no change” to 5 “completely recovered.” A higher score indicates better recovery of the condition) (L. O. P. Costa et al. 2008); Chronic Pain Self‐Efficacy Scale (CPSS) (i.e., 22 items categorised into the 3 domains. Each domain ranges from 10 “a lot of uncertainty” to 100 points “a lot of certainties.” The maximum score of 300 points indicates a higher sense of self‐efficacy) (Salvetti et al. 2005); Quality of life with 12‐item Short form Survey (SF‐12) (i.e., the SF‐12 consists of a subset of 12 items from the SF‐36 Health Survey (SF‐36), where a higher score means better quality of life) (Damásio et al. 2015; Silveira et al. 2013); and Tampa Scale of Kinesiophobia (TSK) (i.e., a 17‐item self‐report checklist using a 4‐point Likert scale, where Higher scores indicate greater kinesiophobia) (De Souza et al. 2008).

### 3.4 Data Analysis

The required sample size was calculated based on detecting a mean difference of 4.2 points on the Neck Disability Index, assuming a standard deviation of 5.5 points, a two‐tailed test, an alpha level of 5%, a desired power of 80%, and an estimated 15% follow‐up loss. We assumed a minimum sample size of 70 participants (35 participants per group). The estimates used in the sample size calculation were based on a previously published study (Domingues et al. 2019).

Participant characteristics were reported through descriptive statistical tests. The treatment effects (i.e., mean differences between groups) and 95% confidence intervals were calculated using mixed linear models with “time versus group” interaction terms. These interaction terms are equivalent to between‐group differences. Missing data were handled using mixed linear models (Twisk 2003). All analyses adhered to the intention‐to‐treat principle (Elkins and Moseley 2015), and the statistical analyses were performed using the Statistical Package for the Social Sciences (SPSS) version 17.0.

## 4 Results

### 4.1 The Flow of Participants Through the Trial

Participants were enrolled between August 2022 and May 2023. Both groups presented comparable baseline characteristics upon entry into the study. The recruitment comprised a total of 70 participants, with 72% being women, averaging 29 years of age, and reporting an average pain duration of 30 months (Table 1).

**TABLE 1:** Baseline characteristics of participants.

Characteristics	All (*n* = 70)	Telerehabilitation (*n* = 35)	Control (*n* = 35)
Age (yr), mean (SD)	29 (8.8)	29.2 (9.0)	28.9 (8.6)
KG, mean (SD)	65.3 (11.7)	64.3 (9.0)	66.4 (14.4)
Height (cm), mean (SD)	1.63	1.63 (0.0)	1.64 (0.0)
BMI (kg/m^2^), mean (SD)	24.2 (3.2)	24.0 (2.7)	24.4 (3.7)
Gender, *n* (%)			
Male	19 (27.1)	10 (28.6)	9 (25.7)
Female	51 (72.8)	25 (71.4)	26 (74.3)
Duration of symptoms (mth), median (IQR)*	30 (15–77)	36 (18–95)	24 (12–60)
Use of medication, *n* (%)	19 (27.1)	9 (25.7)	10 (28.6)
Smoking, *n* (%)	4 (5.7)	3 (8.6)	1 (2.9)
Marital status, *n* (%)			
Single	58 (82.8)	31 (88.6)	27 (77.1)
Married	9 (12.8)	3 (8.6)	6 (17.1)
Divorced	3 (4.2)	1 (2.9)	2 (5.7)
Widowed	—	—	—
Education, *n* (%)			
Elementary school	—	—	—
High school	33 (47.1)	17 (48.6)	16 (45.7)
Higher education	37 (52.9)	18 (51.4)	19 (54.3)
Job, *n* (%)			
Students	21 (30)	11 (31.4)	10 (28.6)
Healthcare professionals	21 (30)	12 (34.3)	9 (25.7)
Office professionals	12 (17.1)	8 (22.8)	4 (11.4)
Labour work	16 (22.9)	7 (20)	9 (25.7)

Abbreviations: mth, months; SD, standard deviation.

**FIGURE 1:**
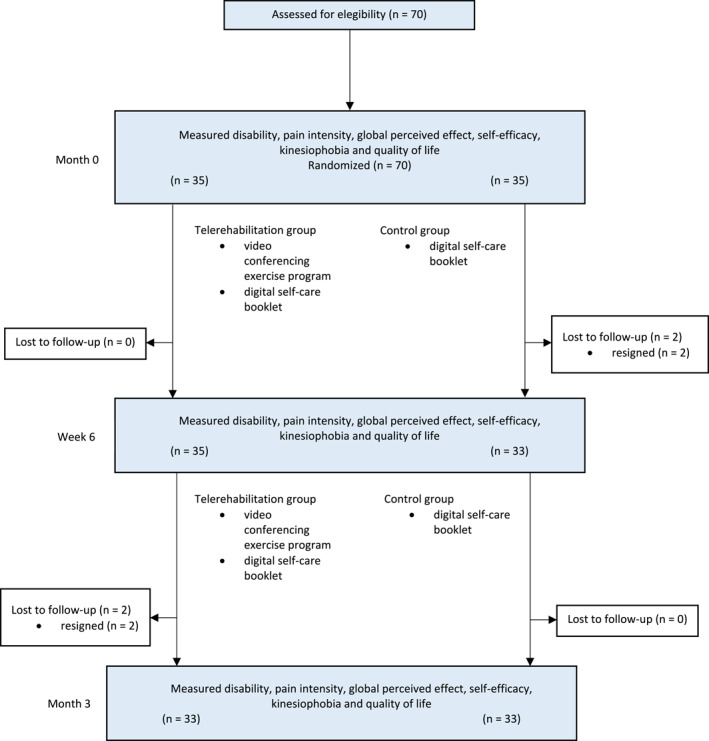
Flow chart of the study.

The flow of participants through the trial and the reasons for the loss of follow‐up of two participants from the experimental group (5%) and two from the control group (10%) are presented in Figure 1.

### 4.2 Effects of Intervention

The results regarding functional disability are presented in Table 2. There was a significant improvement in disability between the groups over 6 weeks, with a noteworthy difference of 10.3 points (95% CI: 4.84–15.79) (Table 2). However, at the 3‐month follow‐up, there was no significant difference between the groups when compared to the baseline, with a difference score of 4.9 points (95% CI: −0.67–10.48) (Table 2).

**TABLE 2:** Mixed linear model analysis for group‐by‐time effect mid‐post intervention.

Outcomes	Difference within groups				Difference between groups	
	6‐weeks		3‐months		6‐weeks 95% (CI)	3‐months 95% (CI)
	Tele	Con	Tele	Con	Tele versus Con	Tele versus Con
Neck disability index (0 a 50)	−14.8 (−19.9 to −9.6)	−4.9 (−10.9 to 1.1)	−12.8 (−18.0 to 7.5)	−8.1 (−13.6 to −2.5)	−10.3 (−4.8 to −15.7)*	−4.9 (0.6 to −10.4)
Pain (0 a 10)	−4.1 (−4.9 to −3.2)	−1.6 (−2.6 to −0.5)	−3.0 (−4.0 to −1.9)	−1.9 (−2.9 to −0.8)	−2.8 (−1.4 to −4.1)*	−1.2 (0.1 to −2.6)
Global perceived effect (0 a 10)	4.4 (3.3–5.4)	2 (0.8–3.1)	4.3 (3.2–5.3)	2.3 (1.2–3.3)	2.3 (3.7 to 0.9)*	2.0 (3.4 to 0.6)*
Self‐efficacy (30 a 300)	38.3 (16.7–59.8)	14.2 (−7.6–35.9)	45.8 (26.3–65.2)	17 (−5.5–39.5)	24.7 (41.0 to 8.4)*	26.3 (42.8 to 9.8)*
Kinesiophobia (17 a 68)	−1.7 (−6.6 to 3.2)	−1.3 (−5.1 to 2.5)	−3.9 (−7.6 to −0.1)	−1.3 (−5.2 to 2.6)	−0.7 (2.3 to −3.8)	−1.4 (1.7 to −4.5)
Quality of life (12 a 56)	2.4 (0.6–4.1)	1.7 (−0.2–3.6)	1.8 (0.1–3.4)	0.4 (−1.5–2.3)	0.7 (2.6 to −1.1)	1.4 (3.3 to −0.5)

*p < 0.05.

For the outcome of pain intensity, there was a reduction in pain intensity, with a significant estimated difference between the groups of 2.80 points (95% CI 1.40–4.19) at 6 weeks. However, no significant differences between the groups were observed at the 3‐month follow‐up (Table 2).

Regarding the global perceived effect outcome, there was a significant improvement at 6 weeks that persisted at the 3‐month follow‐up. The estimated difference between the groups after the 6‐week intervention was −2.38 points (95% CI −3.77 to −0.98). This significant difference between the groups remained at the 3‐month follow‐up, with a difference of −2.00 points (95% CI −3.40 to −0.60).

In the analysis of self‐efficacy, there was a significant improvement between the groups over the 6 weeks, and this improvement persisted at the 3‐month follow‐up. The difference between the groups at 6 weeks was −24.75 points (95% CI −41.09 to −8.41), and at the 3‐month follow‐up, the difference was −26.31 points (95% CI −42.82 to −9.80).

For the outcomes of quality of life and kinesiophobia, there were no significant improvements between the groups at both time points. The data did not yield statistical differences between the groups (Table 2).

## 5 Discussion

This study found that the telerehabilitation programme proposed was effective in reducing functional disability and pain intensity in individuals with chronic non‐specific neck pain after a 6‐week treatment period. These results were not sustained in the 3‐month follow‐up. For the global perceived effect and self‐efficacy outcomes, we found a significant difference for both 6‐week and 3‐month follow‐ups.

While our survey shares some similar findings with the previous studies, it differs in terms of methodology study design, and a diverse range of individual perspectives. To date, there are not many systematic reviews with meta‐analysis that have investigated the effectiveness of telerehabilitation in patients with non‐specific chronic neck pain (Moreno‐Ligero et al. 2023).

A recent systematic review, focussing on evaluating the efficacy of e‐health‐based interventions in addressing chronic LBP symptoms, revealed that e‐health interventions had a discernible impact compared with minimal intervention. Specifically, the review demonstrated a reduction in short‐term intermediate pain intensity (SMD = −0.64, 95% CI: −1.72 to 0.45) and an improvement in short‐term intermediate back‐specific functional status (SMD = −0.39, 95% CI: −0.87 to 0.09). Moreover, these findings were presented with moderate certainty of evidence (Lara‐Palomo et al. 2022).

Similarly our findings, Özel and Kaya Ciddi (2022), reported a significant improvement in pain intensity and functional disability after 4 weeks of treatment for both the supervised and unsupervised telerehabilitation groups, but not for the control group in individuals suffering from chronic neck pain (Özel and Kaya Ciddi 2022). Additionally, Javdaneh et al. (2021) also identified the effectiveness of exercise programme for the cervical region, combined with pain neuroscience education over 6 weeks, showing positive results in reducing pain, disability, pain catastrophizing, and improving self‐efficacy when compared to providing a booklet on proper daily postures (Javdaneh et al. 2021).

Furthermore, similarly our other findings, recent randomised controlled trials aimed to evaluate the effectiveness of holistic exercise and education combination via telerehabilitation on pain, disability, kinesiophobia, exercise adherence, quality of life and patient satisfaction in individuals with chronic neck pain concluded that the telerehabilitation group demonstrated better improvement in function, quality of life (including bodily pain, general health, social function), kinesiophobia (Tejera et al. 2020) and exercise adherence (Özden et al. 2023).

Strengths of our study include methodological rigour, such as randomisation, validated outcome measures, consistent intervention delivery by the same physiotherapist for the intervention group, and pre‐registration of the protocol. Furthermore, telerehabilitation has demonstrated effectiveness in improving neck pain patients through accessible and cost‐effective technologies.

Limitations include the length of follow‐up duration for pain intensity and disability outcomes, which was only 3 months. Therefore, the long‐term sustainability of the observed results remains uncertain.

These results are important and can assist health professionals such as physiotherapists and doctors in recommending treatment for individuals with chronic non‐specific neck pain. Telerehabilitation performed for therapeutic exercises proves to be an effective therapy due to its greater ease of access and low cost for patients with neck pain.

## 6 Conclusion

Telerehabilitation is an effective treatment modality for reducing functional disability and pain intensity, enhancing perceived global effect, and improving self‐efficacy after 6 weeks of treatment when compared to a self‐care pamphlet. Furthermore, it demonstrates efficacy in enhancing perceived global effect and self‐efficacy at the 3‐month follow‐up, in comparison to a self‐care pamphlet. Therefore, telerehabilitation has emerged as an effective treatment alternative for individuals with non‐specific chronic neck pain.

**What was already known on this topic:**

— Neck pain is a common and potentially debilitating condition.
— Telerehabilitation has been established as an effective strategy for various health conditions, including but not limited to lower back pain, osteoarthritis, joint replacements, and chronic musculoskeletal pain. However, its efficacy in the context of non‐specific neck pain remains unclear.

**What this study adds:**

— A 12‐session exercise programme delivered through telerehabilitation compared with educational interventions seems to be effective for people experiencing chronic neck pain.
— The telerehabilitation proved to be effective in improving patients with neck pain through easily accessible and low‐cost technologies.

## Author Contributions

J.C. and M.O. conceived the experiment(s), J.C., B.V. and B.F. conducted the experiment(s), and J.C., B.T. and M.O. analysed the results. All authors reviewed the manuscript.

## Ethics Statement

This study was approved by the Research Ethics Committee (CAAE: 56898122.9.0000.0018). All participants received oral (video conference) and written information about their participation in the trial. Before inclusion, they must accept an informed consent letter.

## Conflicts of Interest

The authors declare no conflicts of interest.

## Data Availability

The data that support the findings of this study are available from the corresponding author upon reasonable request.

## References

[msc70119-bib-0001] Almeida, D. C. , and D. C. Kraychete . 2017. “Dor Lombar ‐ Uma Abordagem Diagnóstica.” Revista Dor 18, no. 2: 173–177. 10.5935/1806-0013.20170034.

[msc70119-bib-0002] Alsobayel, H. , F. Alodaibi , A. Albarrati , N. Alsalamah , F. Alhawas , and A. Alhowimel . 2022. “Does Telerehabilitation Help in Reducing Disability Among People With Musculoskeletal Conditions? A Preliminary Study.” International Journal of Environmental Research and Public Health 19, no. 1: 72. 10.3390/IJERPH19010072. PMC8751178 35010331

[msc70119-bib-0003] Barbosa, J. C. , J. Comachio , A. P. Marques , B. T. Saragiotto , and M. O. Magalhaes . 2023. “Effect of a Telerehabilitation Exercise Program Versus a Digital Booklet With Self‐Care for Patients With Chronic Non‐Specific Neck Pain: A Protocol of a Randomized Controlled Trial Assessor‐Blinded, 3 Months Follow‐Up.” Trials 24, no. 1: 616. 10.1186/S13063-023-07651-Z. 37770963 PMC10537532

[msc70119-bib-0004] Bernal‐Utrera, C. , J. J. González‐Gerez , M. Saavedra‐Hernandez , M. Á. Lérida‐Ortega , and C. Rodríguez‐Blanco . 2019. “Manual Therapy Versus Therapeutic Exercise in Non‐Specific Chronic Neck Pain: Study Protocol for a Randomized Controlled Trial.” Trials 20, no. 1: 487. 10.1186/S13063-019-3598-7. 31399143 PMC6688373

[msc70119-bib-0005] Blanpied, P. R. , A. R. Gross , J. M. Elliott , et al. 2017. “Clinical Practice Guidelines Linked to the International Classification of Functioning, Disability and Health From the Orthopaedic Section of the American Physical Therapy Association.” Journal of Orthopaedic & Sports Physical Therapy 47, no. 7: A1–A83. 10.2519/JOSPT.2017.0302. 18758050

[msc70119-bib-0006] Chan, A. W. , J. M. Tetzlaff , D. G. Altman , et al. 2013. “SPIRIT 2013 Statement: Defining Standard Protocol Items for Clinical Trials.” Annals of Internal Medicine 158, no. 3: 200–207. 10.7326/0003-4819-158-3-201302050-00583. 23295957 PMC5114123

[msc70119-bib-0007] Cohen, S. P. 2015. “Epidemiology, Diagnosis, and Treatment of Neck Pain.” Mayo Clinic Proceedings 90, no. 2: 284–299. 10.1016/J.MAYOCP.2014.09.008. 25659245

[msc70119-bib-0008] Cook, C. , J. K. Richardson , L. Braga , et al. 2006. “Cross‐Cultural Adaptation and Validation of the Brazilian Portuguese Version of the Neck Disability Index and Neck Pain and Disability Scale.” Spine 31, no. 14: 1621–1627. 10.1097/01.BRS.0000221989.53069.16. 16778699

[msc70119-bib-0009] Corp, N. , G. Mansell , S. Stynes , et al. 2021. “Evidence‐Based Treatment Recommendations for Neck and Low Back Pain Across Europe: A Systematic Review of Guidelines.” European Journal of Pain 25, no. 2: 275–295. 10.1002/EJP.1679. 33064878 PMC7839780

[msc70119-bib-0010] Costa, F. , D. Janela , M. Molinos , et al. 2022. “Telerehabilitation of Acute Musculoskeletal Multi‐Disorders: Prospective, Single‐Arm, Interventional Study.” BMC Musculoskeletal Disorders 23, no. 1: 1–12. 10.1186/S12891-021-04891-5. 34983488 PMC8728982

[msc70119-bib-0011] Costa, L. O. P. , C. G. Maher , J. Latimer , et al. 2008. “Clinimetric Testing of Three Self‐Report Outcome Measures for Low Back Pain Patients in Brazil: Which One Is the Best?” Spine 33, no. 22: 2459–2463. 10.1097/BRS.0B013E3181849DBE. 18923324

[msc70119-bib-0012] Damásio, B. F. , T. F. Andrade , and S. H. Koller . 2015. “Psychometric Properties of the Brazilian 12‐Item Short‐Form Health Survey Version 2 (SF‐12v2).” Paideia 25, no. 60: 29–37. 10.1590/1982-43272560201505.

[msc70119-bib-0013] de Campos, T. F. , C. G. Maher , D. Steffens , J. T. Fuller , and M. J. Hancock . 2018. “Exercise Programs May Be Effective in Preventing a New Episode of Neck Pain: A Systematic Review and Meta‐Analysis.” Journal of Physiotherapy 64, no. 3: 159–165. 10.1016/J.JPHYS.2018.05.003. 29908853

[msc70119-bib-0014] De Souza, F. S. , C. Da Silva Marinho , F. B. Siqueira , C. G. Maher , and L. O. P. Costa . 2008. “Psychometric Testing Confirms That the Brazilian‐Portuguese Adaptations, the Original Versions of the Fear‐Avoidance Beliefs Questionnaire, and the Tampa Scale of Kinesiophobia Have Similar Measurement Properties.” Spine 33, no. 9: 1028–1033. 10.1097/BRS.0B013E31816C8329. 18427325

[msc70119-bib-0015] Domingues, L. , F. M. Pimentel‐Santos , E. B. Cruz , et al. 2019. “Is a Combined Programme of Manual Therapy and Exercise More Effective Than Usual Care in Patients With Non‐Specific Chronic Neck Pain? A Randomized Controlled Trial.” Clinical Rehabilitation 33, no. 12: 1908–1918. 10.1177/0269215519876675. 31549519

[msc70119-bib-0016] Elkins, M. R. , and A. M. Moseley . 2015. “Intention‐to‐Treat Analysis.” Journal of Physiotherapy 61, no. 3: 165–167. 10.1016/J.JPHYS.2015.05.013. 26096012

[msc70119-bib-0017] Fandim, J. V. , L. O. P. Costa , T. P. Yamato , et al. 2021. “Telerehabilitation for Neck Pain.” Cochrane Database of Systematic Reviews 2021, no. 3: CD014428. 10.1002/14651858.CD014428. PMC12341027 40792483

[msc70119-bib-0018] Fandim, J. V. , R. Nitzsche , Z. A. Michaleff , L. O. Pena Costa , and B. Saragiotto . 2020. “The Contemporary Management of Neck Pain in Adults.” Pain Management 11, no. 1: 75–87. 10.2217/PMT-2020-0046. 33234017

[msc70119-bib-0019] Fillipo, R. , K. Pruka , M. Carvalho , et al. 2022. “Does the Implementation of Clinical Practice Guidelines for Low Back and Neck Pain by Physical Therapists Improve Patient Outcomes? A Systematic Review.” Implementation Science Communications 3, no. 1: 57. 10.1186/S43058-022-00305-2. 35659117 PMC9164354

[msc70119-bib-0020] Gialanella, B. , T. Ettori , S. Faustini , et al. 2017. “Home‐Based Telemedicine in Patients With Chronic Neck Pain.” American Journal of Physical Medicine and Rehabilitation 96, no. 5: 327–332. 10.1097/PHM.0000000000000610. 27584139

[msc70119-bib-0021] Herbert, R. , G. Jamtvedt , and M. R. E. Kåre Birger Hagen . 2005. Practical Evidence‐Based Physiotherapy. 3rd ed. Elsevier Butterworth Heinemann.

[msc70119-bib-0022] Hoy, D. G. , M. Protani , R. De , and R. Buchbinder . 2010. “The Epidemiology of Neck Pain.” Best Practice & Research Clinical Rheumatology 24, no. 6: 783–792. 10.1016/J.BERH.2011.01.019. 21665126

[msc70119-bib-0023] James, S. L. , D. Abate , K. H. Abate , et al. 2018. “Global, Regional, and National Incidence, Prevalence, and Years Lived With Disability for 354 Diseases and Injuries for 195 Countries and Territories, 1990‐2017: A Systematic Analysis for the Global Burden of Disease Study 2017.” Lancet 392, no. 10159: 1789–1858. 10.1016/S0140-6736(18)32279-7. 30496104 PMC6227754

[msc70119-bib-0024] Javdaneh, N. , A. H. Saeterbakken , A. Shams , and A. H. Barati . 2021. “Pain Neuroscience Education Combined With Therapeutic Exercises Provides Added Benefit in the Treatment of Chronic Neck Pain.” International Journal of Environmental Research and Public Health 18, no. 16: 8848. 10.3390/IJERPH18168848. 34444594 PMC8394804

[msc70119-bib-0025] Kosterink, S. M. , R. M. H. A. Huis in’t Veld , B. Cagnie , M. Hasenbring , and M. M. R. Vollenbroek‐Hutten . 2010. “The Clinical Effectiveness of a Myofeedback‐Based Teletreatment Service in Patients With Non‐Specific Neck and Shoulder Pain: A Randomized Controlled Trial.” Journal of Telemedicine and Telecare 16, no. 6: 316–321. 10.1258/jtt.2010.006005. 20798425

[msc70119-bib-0026] Lara‐Palomo, I. C. , E. Gil‐Martínez , J. D. Ramírez‐García , et al. 2022. “Efficacy of e‐Health Interventions in Patients With Chronic Low‐Back Pain: A Systematic Review With Meta‐Analysis.” Telemedicine and E‐Health 28, no. 12: 1734–1752. 10.1089/TMJ.2021.0599. 35532971

[msc70119-bib-0027] Moreno‐Ligero, M. , J. A. Moral‐Munoz , A. Salazar , and I. Failde . 2023. “mHealth Intervention for Improving Pain, Quality of Life, and Functional Disability in Patients With Chronic Pain: Systematic Review.” JMIR MHealth and UHealth 11: e40844. 10.2196/40844. 36729570 PMC9936365

[msc70119-bib-0028] Özden, F. , M. Özkeskin , İ. Tümtürk , and C. Yalın Kılınç . 2023. “The Effect of Exercise and Education Combination via Telerehabilitation in Patients With Chronic Neck Pain: A Randomized Controlled Trial.” International Journal of Medical Informatics 180: 105281. 10.1016/J.IJMEDINF.2023.105281. 37924590

[msc70119-bib-0029] Özel, M. , and P. Kaya Ciddi . 2022. “The Effectiveness of Telerehabilitation‐Based Structured Exercise Therapy for Chronic Nonspecific Neck Pain: A Randomized Controlled Trial.” Journal of Telemedicine and Telecare. 10.1177/1357633X221095782. 35570728

[msc70119-bib-0030] Russell, T. G. 2007. “Physical Rehabilitation Using Telemedicine.” Journal of Telemedicine and Telecare 13, no. 5: 217–220. 10.1258/135763307781458886. 17697506

[msc70119-bib-0031] Salvetti, M. G. , C. A. M. Pimenta , M. De , G. Salvetti , and C. A. De Mattos Pimenta . 2005. “Validação da Chronic Pain Self‐Efficacy Scale para a língua Portuguesa.” Archives of Clinical Psychiatry 32, no. 4: 202–210. 10.1590/S0101-60832005000400002.

[msc70119-bib-0032] Schulz, K. F. , D. G. Altman , and D. Moher . 2010. “CONSORT 2010 Statement: Updated Guidelines for Reporting Parallel Group Randomised Trials.” BMJ 340, no. 7748: 698–702. 10.1136/BMJ.C332. PMC2844940 20332509

[msc70119-bib-0033] Seron, P. , M. J. Oliveros , R. Gutierrez‐Arias , et al. 2021. “Effectiveness of Telerehabilitation in Physical Therapy: A Rapid Overview.” Physical Therapy 101, no. 6. 10.1093/PTJ/PZAB053. PMC7928601 33561280

[msc70119-bib-0034] Silveira, M. F. , J. C. Almeida , R. S. Freire , D. S. A. Haikal , and A. E. D. B. L. Martins . 2013. “Propriedades Psicométricas do Instrumento de Avaliação da Qualidade de Vida: 12‐Item Health Survey (SF‐12).” Ciência & Saúde Coletiva 18, no. 7: 1923–1931. 10.1590/S1413-81232013000700007. 23827896

[msc70119-bib-0035] Tejera, D. M. , H. Beltran‐Alacreu , R. Cano‐De‐la‐cuerda , et al. 2020. “Effects of Virtual Reality Versus Exercise on Pain, Functional, Somatosensory and Psychosocial Outcomes in Patients With Non‐Specific Chronic Neck Pain: A Randomized Clinical Trial.” International Journal of Environmental Research and Public Health 17, no. 16: 1–19. 10.3390/IJERPH17165950. PMC7460130 32824394

[msc70119-bib-0036] Tuckson, R. V. , M. Edmunds , and M. L. Hodgkins . 2017. “Telehealth.” New England Journal of Medicine 377, no. 16: 1585–1592. 10.1056/NEJMSR1503323. 29045204

[msc70119-bib-0037] Twisk, J. W. R. 2003. Applied Longitudinal Data Analysis for Epidemiology: A Practical Guide. 2nd ed. Cambridge University Press.

[msc70119-bib-0038] Wang, X. , D. J. Hunter , G. Vesentini , D. Pozzobon , and M. L. Ferreira . 2019. “Technology‐Assisted Rehabilitation Following Total Knee or Hip Replacement for People With Osteoarthritis: A Systematic Review and Meta‐Analysis.” BMC Musculoskeletal Disorders 20, no. 1: 1–17. 10.1186/S12891-019-2900-X. 31679511 PMC6825714

[msc70119-bib-0039] Yeo, S. M. , J. Y. Lim , J. G. Do , J. Y. Lim , J. In Lee , and J. H. Hwang . 2021. “Effectiveness of Interactive Augmented Reality‐Based Telerehabilitation in Patients With Adhesive Capsulitis: Protocol for a Multi‐Center Randomized Controlled Trial.” BMC Musculoskeletal Disorders 22, no. 1: 1–9. 10.1186/S12891-021-04261-1. 33902546 PMC8074703

